# Biomarkers of Atrial Fibrillation: Which One Is a True Marker?

**DOI:** 10.1155/2019/8302326

**Published:** 2019-04-01

**Authors:** Pipin Ardhianto, Yoga Yuniadi

**Affiliations:** ^1^Department of Cardiology and Vascular Medicine, Faculty of Medicine, University of Diponegoro, Semarang, Indonesia; ^2^Department of Cardiology and Vascular Medicine, Faculty of Medicine, National Cardiovascular Center Harapan Kita, Universitas Indonesia, Jakarta, Indonesia

## Abstract

Atrial fibrillation (AF) is the most common sustained cardiac arrhythmias and associated with the risk of stroke and death. Continuous development of the diagnostic tool and prognostic stratification may lead to optimal management of AF. The use of biomarkers in the management of AF has been grown as an interesting topic. However, the AF biomarkers are not yet well established in the major guidelines. Among these biomarkers, a lot of data show troponin and brain natriuretic peptides are promising for the prediction of future events. The troponin elevation in AF patients may not necessarily be diagnosed as myocardial infarction or significant coronary artery stenosis, and brain natriuretic peptide elevation may not necessarily confirm heart failure. Troponin T and troponin I may predict postoperative AF. Furthermore, troponin and brain natriuretic peptide gave better prognostic performance when compared with the risk score available today.

## 1. Introduction

Atrial fibrillation (AF) is the most common sustained cardiac arrhythmia in clinical practice and also known as an independent risk factor for stroke [1]. The available data also suggest that AF presence affects deaths up to twofold higher [2]. However, exact mechanism of how AF occurs is not clearly understood. It is hypothesized that structural remodeling and electrical remodeling especially in atrial tissue are the core of initiation and maintaining the AF last longer. Also, changes in the left ventricular geometry due to impairment of left ventricular systolic or diastolic function contribute to atrial remodeling which further maintains AF [3]. The role of pulmonary veins in the mechanism of AF has been described. Isolation of electrical activity of pulmonary veins from atrial tissue is the main goal of AF ablation for many years and showed good efficacy to restore the sinus rhythm, whether by using radiofrequency ablation or cryoballoon in paroxysmal AF and particularly in some persistent AF [4, 5].

Stroke, a catastrophic lifelong risk in AF, substantially becomes central investigation in the management of AF and one of the endpoints in AF clinical trials. Stroke in AF tends to be more severe compared to other stroke origins. Therefore, anticoagulation should be good enough to protect from stroke and simultaneously to not putting in high risk for bleeding. The CHA_2_DS_2_-VASc score is widely used to predict stroke in AF since 2010 [6]. Bleeding risk assessment is an integral part of AF management; HAS-BLED has been widely used to assess bleeding risk in individual [7].

Assessments of biomarkers are widely used in the management of myocardial infarction and heart failure; furthermore, certain biomarkers could largely determine the diagnosis, so they require a special handling with no additional work-up [8–10]. On the contrary, in the guideline for management of AF, the use of biomarkers as part of its management has never been mentioned until in the 2016 European Society of Cardiology guideline for management of atrial fibrillation developed in collaboration with EACTS. Biomarkers were offered a class IIb recommendation with a level of evidence B for further refined stroke and bleeding risk in AF. Biomarkers have been playing significant role as risk predictors and prognostic values of some cardiovascular diseases, but it seems that AF is not one of them due to the lack of information for the use of biomarkers in the management of AF itself [5]. Recently published expert consensus on atrial cardiomyopathy encouraged more data of biomarkers for further understanding the natural history of AF. Histological and electrical change in atrial fibrillation occurred due to multiple factors that include inflammation, oxidative stress, renin angiotensin, growth factor, and ion-channel dysfunction, and even adipose tissue plays a substantial role for the development of AF through its paracrine effect [11]. This review will give information of biomarkers in the development, risk prediction, and prognostic value of the AF according to the recent published studies.

## 2. Cardiac Troponin

Cardiac troponins are regulated for intracellular calcium concentration required in myocardial contraction coupling. Cardiac troponins consist of cardiac troponin I (TnI), troponin T (TnT), and troponin C (TnC). These proteins are integral part of structural proteins involved during sliding interaction of actin thin filaments against myosin thick filaments inside the myocardium. In others, striated muscle has expression of these troponins, but cardiac troponin is coded by a specific gene, and hypothetically, these cardiac troponins have unique characteristics possessed by myocardium. Troponin I and troponin T have high sensitivity and specificity for myocardial damage, unlike troponin C. Detection of these proteins to peripheral circulation always indicates myocardial injury [12]. Given the differences of TnT and TnI, both seem to have a comparable sensitivity and specificity in the diagnostic and prognostic application [13].

Guidelines for myocardial infarction suggest the cutoff points of above 99th percentile of the upper reference limit as the diagnosis of myocardial infarction [8]. To date, high-sensitive troponin can be measured, and thus, progressively slow cardiac disease might have result of slow rise of troponin values. Another thing to consider is the interpretation of troponin levels; one must be careful to interpret the result of positive cardiac troponin as a myocardial infarction per se. Some medical conditions will increase the troponin values enough to detect in peripheral circulation, and this cannot be considered as a traditional myocardial infarction because coronary arterial disease results from plaque rupture. In fact, chronic kidney disease will produce a slow rise level of cardiac troponin chronically [14]. Despite these controversies, additional information of troponin value for clinical management of atrial fibrillation could further help identify future AF and the risk for future events. Furthermore, it appears that even troponin levels that are just above the detection limit significantly influence the cardiac activity even in healthy subjects [15].

Elevated troponin levels and AF might lead to much utilization in clinical practice, to predict the future incidents of postoperative AF (POAF) and to aid the risk stratification in AF. In most studies, overt confounding clinical condition results of elevated troponin were not included (myocardial infarction). Studies on troponin and AF show equivocal result on future AF prediction in perioperative setting, while utilization of troponin for the future risk stratification in AF patients still gives the optimism.

Potential link between AF, abnormal ventricular perfusion, and myocyte dysfunction resulting in troponin release is mediated by the renin angiotensin-aldosterone system. AF increases cardiac tissue angiotensin II levels which further cause oxidative stress that may impair the ventricular microvascular bed resulting in ischemia and myocardial dysfunction [16, 17]. Goette et al. [18] tried to explore possible mechanism between rapid atrial activations like AF and troponin elevation. Rapid atrial pacing up to 600 bpm was performed in a total of 14 pigs, 5 of them in the presence of angiotensin II type 1 receptor inhibitor (irbesartan), and 5 of them without irbesartan and the rest of the pigs served as a sham (no intervention). This study has shown that rapid atrial pacing will increase oxidative stress mediated by angiotensin II, as seen in the group without irbesartan which has high NADPH, low-density lipoprotein receptor-1 (LOX-1), and ventricular F2-isoprostane levels compared to the group with irbesartan. Furthermore, oxidative stress impaired myocardial blood flow, resulting in troponin elevation [18]. Other possible mechanisms linking between AF and ventricular dysfunction are mediated through nitric oxide (NO) pathway. A study shows that rapid and persistent atrial pacing increases asymmetric dimethylarginine (ADMA), which is an inhibitor for endothelial nitric oxide synthase inhibitor (eNOS). NO metabolism influences ventricular performances, by which NO could reduce ventricular remodeling [19].

### 2.1. Troponin and Prediction of Postoperative AF

Leal et al. [20] studied a retrospective cohort of 95 patients admitted to hospital for nonemergency coronary artery bypass graft procedure. These researchers divided patients into two groups based on the occurrence of AF and found 25 of patients experienced AF during hospitalization. In this study, AF was defined as any occurrence of AF within hospitalization period that required medical treatments and/or lasting for more than 20 minutes. Troponin I was taken before CABG and postoperatively at ICU admission. The postoperative troponin level was lower in the sinus rhythm group compared to the AF group (0.66 ± 1.66 vs 2.07 ± 5.01 ng/ml, *p*=0.029). The ROC was 0.901 ng/ml, which was the best cutoff value for predicting AF within hospitalization. The calculated area under curve was 0.71 (95% CI, 0.57–0.83). Odds ratio was 11.5 (95% CI, 3.8–34.8) for occurrence of AF after CABG within hospitalization [20].

Leal et al. conclude that the TnI level above 0.901 ng/mL distinguished the low risk group from the group at high risk for AF development perioperatively. The proposed mechanism mentioned by Leal et al. was multifactorial and may be linked to heart manipulation, myocardial ischemia, atrial distention, inflammatory process, and previous structural disease [20].

Narducci et al. [21] studied further regarding the role of inflammation or myocardial ischemia in the development of postoperative AF (POAF). The inflammation marker was reflected by peripheral blood samples of hsCRP and atrial biopsies before CABG to obtain immunohistochemistry of CRP, while myocardial ischemia was reflected by hs-TnT. They studied 38 patients who underwent CABG of which 14 of them experienced POAF. The hsCRP and atrial CRP did not correlate with POAF. The postoperative level of hs-TnT was higher in the POAF group compared to the SR group with median level 0.52 vs 0.30 ng/mL, respectively, with *p* value = 0.016. This study suggested myocardial ischemia during surgery has an important role in the development of POAF compared to systemic or local (atrial) inflammation [21].

As the authors stated above, the role of troponin as predictors for POAF is equivocal. Knayzer et al. [22] carried out a prospective research of 156 consecutive patients to examine the relation between inflammation associated parameters to postoperative TnI and POAF. This study found 50 patients who had POAF. The first episode of AF occurred between the day of surgery and postoperative day 6. Mean duration of AF was 21.8 ± 8.1 hours and median 9 hours. Univariate analysis did not find any association between postoperative TnI and POAF occurrence and further in multivariate analysis found only LA enlargement and prolonged hospital stay independently associated with POAF.

Masson et al. performed a study as the ancillary of the Omega-3 Fatty Acids for Prevention of Post-operative Atrial Fibrillation (OPERA) multicenter randomized trial [23]. The uncertain role of biomarkers in POAF boosts the importance of this study. The primary end point was POAF occurrence of at least 30s. High sensitivity troponin T (hs-TnT) was taken serially: at enrollment, on the morning of surgery, at the end of surgery (skin closure), and on day 2 after surgery. From univariate analysis, the POAF group had a higher hs-TnT level compared to no-POAF groups (14.5 vs 11.0, *p*=0.002, showed as median). hs-TnT showed linear associations with POAF risk until 27 ng/mL, with no additional increase risk thereafter. From multivariate analysis, hs-TNT failed to show the association with the risk of POAF. Relative changes in hs-TnT levels in the morning and at the end of surgery were not associated with the risk of POAF. Furthermore, subgroup analysis (defined by demographic, clinical, and surgical characteristics) still showed no association between hs-TnT and risk for POAF.[23].

To our knowledge, a study with the largest sample sizes in accordance with troponin and POAF was performed by Koolen et al. [24]. These researchers prospectively studied 3148 patients undergoing CABG, and three fixed-time TnT values were measured: before surgery, arrival at ICU, and 8–12 h later on. POAF occurred in 1080 patients (34%); TnT at ICU and TnT at 8–12 h after ICU admission were significantly higher in POAF groups compared to no-POAF groups (0.44 ± 1.07 vs 0.34 ± 0.66 ng/mL, *p* ≤ 0.001 and 0.67 ± 1.66 vs 0.45 ± 0.96 ng/mL, respectively, *p* ≤ 0.001). Multivariate analysis, once again, failed to show independent association of TnT and POAF, and furthermore, ROC analysis demonstrated no cutoff points could be established for prediction of POAF.

Masson et al. [23] and also Koolen et al. [24] considered the lack of independent predictive ability of troponin for POAF prediction as consequence of complex interplay between preexisting conditions and surgical stress in triggering POAF. Troponin alone seems enough for POAF prediction in univariate analysis, but when aligned with other variables responsible for POAF, it will become weak. All the data supporting the hypothesis for POAF mechanism which is multifactorial include [1] patient's factors such as age, atrial dilation, structural changing of the heart, and other comorbid conditions [2] and surgical procedures such as operative trauma, pericardial lesion, use of catecholamines, electrolyte imbalance, and parasympathetic activation [25].

### 2.2. Troponin and Risk Stratification in AF

Troponin elevation is almost always related to acute coronary syndromes (ACS). High sensitivity for ACS of these assays should prompt to search other etiologies when facing with nonischemic-related symptoms. In cases of tachycardia, small case series of patients showed convincingly elevation of troponin I in supraventricular arrhythmia including AF with normal epicardial coronary arteries [26]. Underlying mechanisms proposed are diastolic time shortening and subendocardial ischemia. Majority of coronary perfusion occurred during diastole; shortened diastolic time in tachycardia attenuated the blood flow, resulting in ischemia. Another possible mechanism is myocardial stretch during tachycardia showed by direct relationship between NT-pro BNP and troponin in various types of tachycardia [27].

Emergency setting of patients with symptomatic AF, ST-segment depression on ECG, and no clear ischemic symptoms with troponin elevation made a diagnosis confusion for the presence of significant coronary artery disease (sCAD). The lack of troponin to predict significant coronary artery disease requiring intervention in AF was found in several prospective studies of acutely symptomatic patients of AF in the emergency setting [27–29]. Parwani et al. [29] found that AF patients with a high rate and/or ischemic symptoms frequently associated with TnI elevation presented in the emergency. In this setting, TnI had a poor predictive value for significant coronary artery disease. It seems that TnI elevation was influenced by the heart rate at presentation. Similar findings were found in a larger study by Alghamry et al. Initial elevation of TnI was not correlated with sCAD (OR 0.99, 95% CI 0.79–3.32, *p*=0.19). On serial TnI measurement, peak TnI was correlated with sCAD [28].

Acute setting of AF presentation at the emergency department usually had a faster heart rate. Several studies showed that the faster heart rate is one of the determinant factors for elevated TnI. This elevation did not have a strong association with significant epicardial coronary artery disease suggested by several studies. Tachycardia could be the point of ischemic stress in this clinical setting as researchers found on multivariate analysis [28–30]. This finding suggests that troponin elevation alone cannot be directly considered as myocardial infarction even though the troponin level increased above 99th percentile as the guideline recommendation. Diagnosis assessment made at the emergency department can prevent such patients from administering unnecessary drugs. Patients with AF have already taken medication for anticoagulation, when additional dual antiplatelets are added which will increase the risk for bleeding. It seems that, in this clinical condition, certain criteria are needed to make diagnosis of the presence or absence of myocardial infarction clear because the use of 99th percentile elevation of troponin seems to be useless.

Stroke risk stratification is an integral part in the assessment of AF. The CHADS_2_ score is based on clinical factors such as congestive heart failure, hypertension, age above 75 years, diabetes mellitus, and previous stroke or transient ischemic attack. The CHA_2_DS_2_VASc was made as a modification of the previous risk score. The major improvements were the application of two points for previous stroke, age above 75 years, and transient ischemic attack and one point for clinical risk factors (heart failure, hypertension, diabetes mellitus, vascular disease, female gender, and age) [5]. CHA_2_DS_2_VASc performed better than CHADS_2_ in a real-world validation scheme, especially to describe the truly low risk group. However, when the CHA_2_DS_2_VASc score of 1 or above did not have the equal risk among the specific covariates composing the score, the risk factor of previous thromboembolism is considered the highest risk factor among others [31, 32]. Recently, a concept of delta CHA_2_DS_2_VASc score has been introduced to better stratify risk of stroke, reflecting the dynamic change in scores between baseline and follow-up, which was strongly predictive of ischemic stroke [33].

Association of adverse outcome and AF had been identified by van den Bos et al. [34] that found the correlation of minor elevation of troponin in AF hospitalized patients and adverse events. In a multivariate model, minor troponin I elevation and a positive troponin I were independently associated with death (HR: 2.35, 95 % CI: 1.17–4.73 for minor elevation and HR: 3.77, 95% CI: 1.42–10.02 for positive troponin). These researchers proposed 5 mechanisms by which troponin is released during AF: (1) fast ventricular response can cause demand ischemia in normal coronary arteries, (2) coronary blood flow was reduced during AF, (3) coexistence with significant coronary artery disease, (4) increase in LV wall stress in AF, and (5) acute thrombotic events with preexisting AF. These are common findings.

A substudy from “Randomization Evaluation of Long-Term Anticoagulant Therapy” (RE-LY) covering 6189 patients investigated the prevalence of elevated TnI and its association to cardiovascular events including stroke and death. TnI levels were extremely skewed to the left as expected. Detectable TnI was found in 57% patients and elevated TnI in 24.6% patients. Proportion of the CHADS_2_ score was significantly correlated to TnI levels. A low CHADS_2_ risk score (0–1) was significantly prevalent in the undetectable and lower TnI level, while a high CHADS_2_ risk score (≥3) was significantly prevalent in the elevated TnI level. The interesting findings are the pattern of gradually higher rates of thromboembolic end point concomitant with higher troponin levels correlated to in all CHADS_2_ scores, including with CHADS_2_ score 0–1. The highest annual rates of thromboembolic end point of 11.4% were found in high CHADS_2_ score ≥3 and highest TnI levels compared to lowest annual risk of 1.48% in the CHADS_2_ score 0–1 and undetectable TnI. The annual vascular death rate was 1.04% in comparison with 6.56% (HR, 4.38; 95% CI, 3.05–6.29) in the highest TnI group. Furthermore, the annual rate of major bleeding was significantly higher in the highest TnI levels compared to undetectable TnI levels. Once again through this big study, the TnI level had a high prevalence in AF patients. The degree of TnI levels independently associated with raised risk of stroke or systemic embolism, mortality, and other cardiovascular events [35].

 Hijazi et al. [35] emphasize on addition of TnI value to low CHADS2 score for AF risk stratification. In the low-risk CHADS_2_ score, addition of increase TnI ≥0.020 *µ*g/L doubled the risk of stroke, while any increase ≥0.040 *µ*g/L raised the risk of stroke up to 5-fold, surpassed the annual risk for a high CHADS_2_ score. Clinical implication of this group of patients required specific assessment and anticoagulants for stroke prevention as in patients with a high CHADS_2_ score. Similar findings were shown in relation to the low CHA_2_DS_2_VASc score. In a group of patients with a high CHADS_2_ score and elevated TnI, there was a residual risk of stroke even in the anticoagulant therapy which further advised to intensify the upstream therapy for neurohumoral activation, AF ablation, left atrial appendage closure, left atrial volume reduction [35]. Consistent results were found in high-sensitivity TnT in association with adverse outcome in AF. The high-sensitivity TnT level in AF boosts prognostic performance to predict stroke combined with clinical characteristics established earlier [36, 37].

### 2.3. B-Type Natriuretic Peptide

A normal heart secretes hormones in atrial tissue as a response to regulate fluid hemostasis and blood pressure. Atrial natriuretic peptide (ANP) and B-type natriuretic peptide (BNP) are secreted in the response to atrial distention. Integration of biomarkers in heart failure including B-type natriuretic peptide (BNP) as well as N-terminal BNP (NT-pro BNP) is part of many studies in the diagnosis and prognosis assessment. Development of biomarkers in the past decade shows that BNP is used in a variety study purposed for both diagnosis and prognosis of heart failure [38, 39]. The 2016 ESC Guidelines recommends BNP of 35 pg/mL and NT-pro BNP of 125 pg/mL as diagnostic cutoff value for heart failure [9].

In case of AF, BNP elevation was firstly claimed by Silvet et al. in 72 stable outpatients with AF. AF patients had significantly higher BNP levels compared to the normal subject, and the median BNP level was 131 pg/mL [40]. This BNP value can be considered as a heart failure based on the recent guideline published [9]. A substudy from BNP for Acute Shortness of Breath Evaluation (BASEL) evaluated the use of BNP in the management of acute dyspnea with AF. These researchers highlighted some findings, but the most interesting was AF with stable HF had an increased level of BNP such that the diagnosis of heart failure with AF need a higher level of BNP compared to heart failure without AF [41].

A high BNP or NT-pro BNP level in AF without sign of significant heart failure invites area for research. Presence of AF impaired the diagnostic performance of AF. Richards et al. found a diagnostic challenge in patients presenting with acute dyspnea and AF. The BNP level interrupting heart failure is a cause of acute dyspnea [42]. In AF patients presenting with heart failure, it seems reasonable to put in higher BNP levels ≥ 500 pg/mL since the BNP level in AF and heart failure was comparable to heart failure without AF [41, 43]. It seems obvious that, in AF, the BNP or NT-pro BNP level was increased but the exact mechanism is not yet fully gained. Some studies support this finding, so that in AF patients, atrial stretch could therefore increase BNP level [39].

In the RE-LY substudy carried out by Hijazi et al., it was found that NT-pro BNP significantly associated with age, AF, history of congestive heart failure, and lower creatinine clearance. NT-pro BNP was directly correlated with CHADS_2_ score, that is, lower NT-pro BNP level associated with smaller CHADS_2_ score. Adverse outcome also occurred based on the NT-pro BNP level aside the troponin level discussed above including thromboembolic events; furthermore, on multivariate analysis NT-pro BNP was still associated with adverse outcome. But, NT-pro BNP was not associated with bleeding risk [35].

An increased level of NT-pro BNP was prevalent in 14892 patients recruited in the substudy from Apixaban for the Prevention of Stroke in Subjects with Atrial Fibrillation (ARISTOTLE) trial. The median level of NT-pro BNP was 715 ng/mL, and first quartile was closer to healthy subjects. Univariate and multivariate analyses showed that clinical characteristics such as age, female sex, diabetes, renal dysfunction, and most strongly the AF type were associated with NT-pro BNP. The highest annual rate of stroke and systemic embolism (2.45%) was found in the group with CHA_2_DS_2_VASc score 3 and NT-pro BNP level >1250 ng/L compared to the averaged annual rate of stroke 0.56% in CHA_2_DS_2_VASc < 2 and level of NT-pro BNP < 363 ng/L. In contrast to other major outcomes, in bleeding risk assessment, NT-pro BNP failed to show its association [44]. Consistent results of NT-pro BNP to predict stroke in these 2 large prospective trials added future optimism of biomarker utilization in AF. The compilation of studies in AF biomarkers is listed in Table 1 [35, 44].

## 3. Renin Angiotensin System

There are some evidence to suggest that the renin angiotensin system (RAS) is associated with the development of AF in subjects with systemic hypertension and heart failure [45, 46]. Furthermore, multiple RAS gene polymorphisms have been linked to the development of AF in subjects with known conditions that directly or indirectly result in increased left atrial pressure, such as systemic hypertension or heart failure [47]. In addition, human atrial myocyte analysis during cardiac surgery showed increased tissue levels of angiotensin-converting enzyme (ACE) and angiotensin II (AT-II) receptors in subjects with AF compared to those with sinus rhythm [48]. The activation of RAS results in electrical and ultrastructural changes, called “atrial remodeling,” which is thought to play a role in the development of AF in humans. Animal models of rapid atrial pacing-induced AF have shown high atrial tissue levels of ACE, chymase, and angiotensinogen. Increased production of tissue-level AT II mediated by paracrine activation of ACE, chymase, and angiotensinogen is also thought to be responsible for atrial remodeling leading to AF [46].

Some clinical trials on RAS antagonism were conducted for primary prevention of AF in the setting of hypertension, heart failure, and coronary artery disease. However, the results are conflicting; some trials showed benefit of angiotensin-converting enzyme inhibitor (ACEI) and angiotensin receptor blocker (ARB) to reduce AF occurrence, but others showed no effect [49, 50]. Similar conflicting results are shown in secondary prevention of AF. Some major trials such as J-RHYTHM II and ANTIPAF failed to show benefit of RAS antagonism for secondary prevention of AF [51, 52]. The role of ACEI and ARB in prevention of AF recurrence in normal and hypertensive patients has been elaborated when they were administered in combination with amiodarone [53, 54].

## 4. Future Direction

Recently published risk score had mentioned biomarker utilization to predict future stroke and bleeding events, namely, ABC (age, biochemistry, and clinical history) stroke risk score, and ABC bleeding risk score. In the 2016 ESC Guidelines for the management of atrial fibrillation developed in collaboration with EACTS, the ABC stroke risk score was given class IIb recommendation [5]. This was internally validated from 14701 patients in Apixaban for Reduction in Stroke and Other Thromboembolic Events in Atrial Fibrillation (ARISTOTLE) trial and externally validated from 700 patients in Stabilization of Atherosclerotic Plaque By Initiation of Darapladib Therapy (STABILITY) trial. The ABC stroke risk score was developed with purpose of using new biomarkers-based risk score to improve prognostic of stroke in AF. The most important predictors for stroke are previous stroke or TIA, which was consistent with many studies, followed by NT-pro BNP, cardiac high-sensitivity troponin, and age. The ABC risk score had higher *C*-statistic indices compared to widely used CHA_2_DS_2_VASc for both internal (0.68 vs 0.62, *p* < 0.001) or external validation (0.66 vs 0.58, *p* < 0.001) [55].

ABC bleeding risk score comprises 3 biomarkers (hemoglobin, growth differentiation factor-15, and hs-troponin). ABC bleeding risk also performed better with high *C*-statistic compared to HAS-BEED (0.68 vs 0.61, *p* < 0.0001). NT-pro BNP was not included in this ABC bleeding risk score since from the RE-LY substudy, NT-pro BNP failed to show the association with bleeding in AF [35, 56]. Integration of biomarkers in risk prediction is promising since the ABC stroke risk score and ABC bleeding risk score performed better compared to guideline recommendation. ABC stroke risk score is simple, there are only 4 parameters needed: age, hs-troponin, NT-pro BNP, and history of stroke/TIA [55]. However, routine usage of ABC stroke risk score and/or ABC bleeding risk score in the routine daily practice are discouraged until more data are available.

## 5. Conclusion

In the past decade, studies on biomarkers in AF patients are increasing. The troponin and BNP or NT-pro BNP are among biomarkers extensively studied recently and being used as part of stroke and bleeding risk assessment in AF. Some data provide superior results of troponin and brain natriuretic peptide when compared to the risk score recommended by the guideline. However, further studies are warranted to confirm the biomarkers position in determining stroke risk and bleeding risk in AF patients.

## Figures and Tables

**Table 1:** Main clinical study concerning the use of biomarkers in AF.

References	Design	Main findings
Knayzer et al. [22]	Prospective study of 156 consecutive patients who underwent isolated coronary artery bypass surgery	(i) Significant correlation between clinical markers of inflammation and post-cardiac surgery elevation in plasma cTnI levels (ii) No correlation between markers and postoperative AF, and there was no correlation between postoperative plasma TnI levels and the occurrence of AF

Masson et al. [23]	Prospective study of 562 patients was performed with serial NT-pro BNP and hs-troponin measurement from randomized to perioperative supplementation with oral fish oil or placebo in the Omega-3 Fatty Acids for Prevention of Post-operative Atrial Fibrillation (OPERA) trial	(i) Univariate analysis; POAF group had higher hs-TnT level vs no-POAF groups. hs-TnT showed linear associations with POAF risk until 27 ng/mL, with no additional increase risk thereafter (ii) Multivariate analysis; both markers failed to show the association to the risk of POAF

Koolen et al. [24]	Retrospective study of prospectively collected data. 3148 patients undergoing elective CABG were evaluated. Serial troponins were measured.	(i) Perioperative TNT is univariably associated with postoperative AF after CABG, but not independently (ii) Further, no clinically useful cutoff point for preventive or early treatment could be identified

van den Bos et al. [34]	Prospective study of 407 patients admitted to the cardiology ward or coronary care unit with atrial fibrillation. TnI was measured serially	(i) Minor troponin I elevation was independently correlated to death, MI

Hijazi et al. [35]	Randomized controlled trial with 6,189 patients from Randomized Evaluation of Long-Term Anticoagulation Therapy (RE-LY) trial	(i) Proportion of CHADS_2_ score was significantly correlated with TnI levels (ii) TnI levels correlated with adverse event of stroke and vascular mortality (iii) Biomarkers increased the *C*-statistic from 0.68 to 0.72, *p*=0.0001, for a composite of thromboembolic events

Roldán et al. [36]	Cohort study with 930 patients, permanent AF, and good anticoagulation control with stabile INR values for at least 6 months (INRs, 2.0–3.0; time in therapeutic range (TTR), >70%)	TnI was associated with combination of stroke, TIA, systemic embolism, acute coronary syndrome, acute heart failure, and cardiac death

Silvet et al. [40].	Prospective study of 72 outpatients with AF and 49 control patients without AF	First study that has shown BNP levels to be significantly elevated in male and female outpatients with chronic AF compared with patients in sinus rhythm

Hijazi et al. [44]	Randomized control trial of 14,892 patients from Apixaban for the Prevention of Stroke in Subjects with Atrial Fibrillation (ARISTOTLE) trial	(i) NT-pro BNP level is elevated in the majority of patients with persistent or permanent AF (ii) Median level of NT-pro BNP was 715 ng/mL. (iii) NT-pro BNP improves risk stratification beyond the CHA_2_DS_2_VASc score (iv) NT-pro BNP was not associated with bleeding risk

AF: atrial fibrillation; TnI: troponin I; TnT: troponin T; MI: myocardial infarct; BNP: brain natriuretic peptide; NT-pro BNP: N-terminal BNP (NT-pro BNP); POAF: postoperative atrial fibrillation; CABG: coronary artery bypass graft.
